# Modeling of Laser-Induced Plasmon Effects in GNS-DLC-Based Material for Application in X-ray Source Array Sensors

**DOI:** 10.3390/s21041248

**Published:** 2021-02-10

**Authors:** Alexander N. Yakunin, Sergey V. Zarkov, Yuri A. Avetisyan, Garif G. Akchurin, Nikolay P. Aban’shin, Valery V. Tuchin

**Affiliations:** 1 Laboratory of System Problems in Control and Automation in Mechanical Engineering, Institute of Precision Mechanics and Control, RAS, 410028 Saratov, Russia; 2 Laboratory of Laser Diagnostics of Technical and Living Systems, Institute of Precision Mechanics and Control, RAS, 410028 Saratov, Russia; szarcov@gmail.com (S.V.Z.); yuaavetisyan@mail.ru (Y.A.A.); akchuringg@mail.ru (G.G.A.); tuchinvv@mail.ru (V.V.T.); 3 Department of Optics and Biophotonics, Saratov State University, 410012 Saratov, Russia; 4 Volga-Svet Co., Ltd., 410052 Saratov, Russia; npabanshin@mail.ru; 5 Interdisciplinary Laboratory of Biophotonics, Tomsk State University, 634050 Tomsk, Russia

**Keywords:** X-ray biosensor, hybrid material, gold nanostar, diamond-like carbon, surface plasmon effect, photoinduced electron emission, wavelength dependence, hot electron, X-ray source

## Abstract

An important direction in the development of X-ray computed tomography sensors in systems with increased scanning speed and spatial resolution is the creation of an array of miniature current sources. In this paper, we describe a new material based on gold nanostars (GNS) embedded in nanoscale diamond-like carbon (DLC) films (thickness of 20 nm) for constructing a pixel current source with photoinduced electron emission. The effect of localized surface plasmon resonance in GNS on optical properties in the wavelength range from UV to near IR, peculiarities of localization of field and thermal sources, generation of high-energy hot electrons, and mechanisms of their transportation in vacuum are investigated. The advantages of the proposed material and the prospects for using X-ray computed tomography in the matrix source are evaluated.

## 1. Introduction

Improvement of breast digital computed tomography (DCT) systems designed for the monitoring and early diagnosis of cancer is one of the most relevant areas of development of high-tech technologies in the interests of modern medicine [1]. The two main technical limitations of modern DCT technology are low spatial resolution and long scanning time [2]. Therefore, a large amount of research [3,4,5,6,7,8,9,10,11,12] has aimed to replace devices with a rotating or moving X-ray tube with matrix radiation sources. The use of such arrays allows one to provide a double positive effect. Firstly, the source matrix is activated without mechanical movement, but by switching the control potentials. Therefore, the speed of scanning and generating projection images necessary for the synthesis of a three-dimensional image of the object under study increases. An important role is played by the efficiency of algorithms for digital processing of a large data array [13]. Secondly, eliminating the mechanical motion of the X-ray source due to radiation eliminates image blur and increases spatial resolution. Reduction in the size of the elementary current source (within the pixel) also contributes to improvement of the quality of the X-ray image. This aspect has determined the orientation of many researchers towards the development of matrix emitters with field electron emission [3,4,5,6,8,9,10,11], which do not require the use of bulky high-temperature heaters. In almost all of these structures, carbon nanotubes were used as sources of field emission. Only the authors [5] synthesized an array of emitters based on tungsten trioxide (WO_3_ NW) nanowires with artificially formed defects to increase the field emission current density to about 14 mA/cm^2^.

It should be noted that the problem of ensuring the reliability and durability of field emission emitters is fundamental because of the inherent exponential dependence of the emission current density on the strength of the local electrostatic field. The result of such criticality may be the spontaneous development of the accelerating thermal breakdown at small voltage fluctuations, which leads to irreversible degradation of the emission properties and, ultimately, to the destruction of the emitter [14]. In ref [15], a composite structure of the blade type with field emission was described. The results of our experimental study showed a durability of 8700 h with an average emission current density of at least 100 mA/cm^2^. Additionally, high photosensitivity of the structure in a wide wavelength range was found in our experiments [16,17,18].

We propose to use the phenomenon of localized surface plasmon resonance (LSPR) of metal nanoparticles, which has become widespread in numerous biomedical applications related to imaging, therapy, and diagnostics [19]. The main manifestation of LSPR is the resonant absorption of the irradiating optical field caused by collective oscillations of equilibrium (Drude electrons) and nonequilibrium (hot) electrons. High-energy hot electrons generated on the surface of plasmon nanoparticles [20,21,22] play a key role in a number of practically important processes: hot electrons can interact with molecules on a surface and induce photochemistry (for example, photochemical water splitting [23]); and hot electrons can enhance and tailor photoelectron emission in Schottky-barrier “metal-semiconductor” photodetectors and solar cells [24]. Perhaps the most interesting thing for us is the possibility of hot electrons to activate the photoemission of electrons from a metal surface into a vacuum. It is these issues of effective generation of hot electrons for the activation of blade-type photoemission structures in a strong electrostatic field to which our theoretical and experimental studies were devoted [16,17,18].

As a development of pixel current sources with photoinduced electron emission, this paper describes a new hybrid material (HM) based on gold nanostars (GNS) embedded in a nanoscale film of diamond-like carbon (DLC). The effect of GNS on optical properties in the wavelength ranging from UV to NIR, peculiarities of localization of field and thermal sources, generation of high-energy hot electrons, and mechanisms of their transportation into a vacuum are investigated. The advantages of the proposed material and the prospects for its use in the matrix source of the X-ray computed tomography sensors are evaluated.

## 2. Features of the Processes of Tunnel Photoemission into Vacuum

In this work, we consider the processes associated with the generation and tunneling of hot electrons at the interface “nanoparticle–vacuum” or “semiconductor film–vacuum” in a strong external electrostatic field. This differs them from most of the works [20,21,22] devoted to the problems of solar energy conversion or catalysis. The motivation for the work was previous encouraging results [25,26], as well as our experimental data [14,15,16,17,18,27,28] on tunneling emission of hot electrons from metal (Mo) and composite structures such as a “metal–DLC (α-C) film” in a vacuum under conditions of combined exposure to a strong electrostatic field and laser radiation. It was found that irradiation of interdigital structures such as the “metal–DLC film” of a vacuum microcathode with CW laser radiation of milliwatt power at discrete wavelengths from 405 to 1550 nm produced a tunnel photocurrent [27], which linearly depended on the light intensity. With an elevation of the voltage at the anode, an exponential increase in the photocurrent was observed, which is characteristic for tunneling emission. Similar experiments on the emission into a vacuum of hot electrons from a metal and the above-mentioned composite were also carried out under irradiation with nanosecond laser pulses of the mJ level in the range 400–1200 nm [28]. It is characteristic that the red threshold of the photoelectric effect for Mo exceeded the photon energy of the laser beam by a factor of 4–6, which indicated precisely the tunneling mechanism of emission.

**Figure 1:**
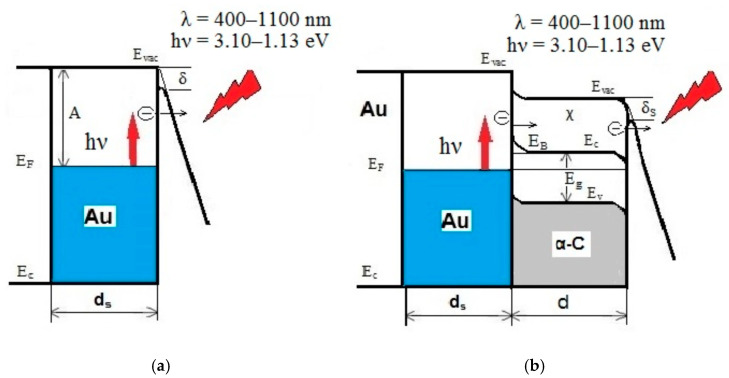
Diagram of the energy levels of a gold nanoparticle (**a**) and a hybrid material—gold nanostars (GNS), embedded into a diamond–like carbon (DLC) film (**b**) in a strong electrostatic field *F* under irradiation with light in the visible or NIR range.

In an HM, which is GNS embedded in a DLC film, in the presence of a strong electrostatic field, two electron transport mechanisms are implemented, as shown in Figure 1. For “metal–DLC film” structures, an additional potential barrier appears at the metal–semiconductor, as well as the process of drift transfer of nonequilibrium charges in the DLC film and their subsequent tunneling through the potential barrier at the “semiconductor–vacuum” interface, as shown in Figure 1b.

In Figure 1 and further, we used the following designations and values: spectral range was λ = 400–1100 nm; the energy of nonequilibrium “hot” electrons corresponded to the indicated wavelength range −3.10–1.13 eV; *E_F_* was the Fermi level; *E*_VAC_ was the vacuum level; *E*_g_ was the width of the forbidden zone in the DLC; *E_C_* and *E_V_* were the energy levels corresponding to the bottom of the conduction band and the top of the valence band in the DLC; χ was the electronic affinity; *E*_B_ was the height of the potential barrier at the metal–semiconductor interface; δ = (*e*^3^β*F*)^1/2^ ≈1.2 (*F*(V/nm))^1/2^ was the decrease in the height of the potential barrier in a strong electrostatic field at the metal–vacuum interface (see Figure 1a) taking into account the Schottky effect and δ_s_ for a semiconductor film with reduced field strength *F* in the film by a factor of ε; ε was the dielectric constant of the film material, β was the form factor of strengthening the electrostatic field due to the curvature of the surface at the semiconductor–vacuum interface; work function A = *E*_VAC_ − *E*_F_; the shape of the potential barrier at the metal–vacuum interface was determined by the ratio (*eF*ζ − *e*^2^/4ζ), where *F* is the strength of the electro-static field, *e* is the electron charge, ζ is the distance from the interface; *d*_s_ was the thickness of the skin layer (on the order of several tens of nanometers for noble metals); and *d* was the thickness of the diamond-like nanofilm (10–30 nm).

**Figure 2:**
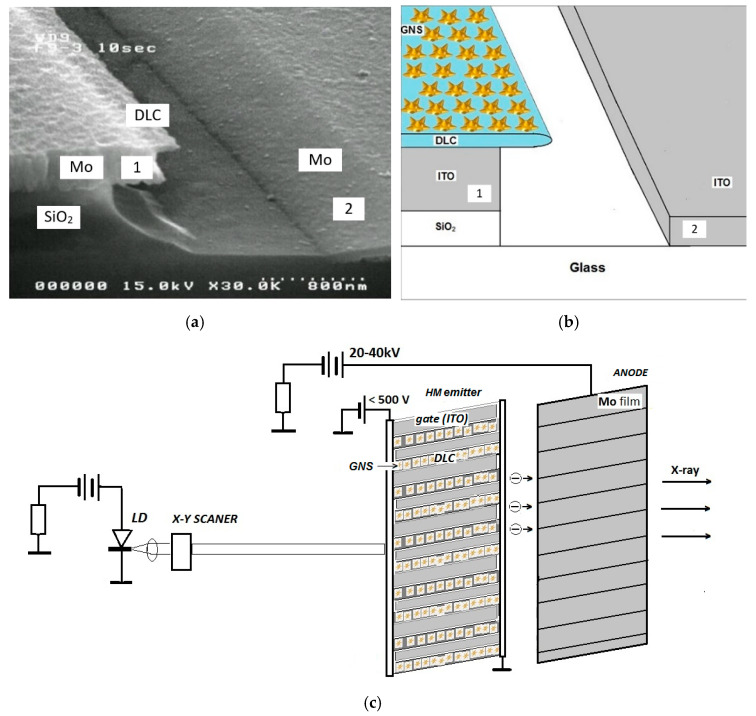
SEM image of a previously developed emitter in the form of a Mo–DLC blade [18] (**a**); schematic representation of the proposed hybrid nanostructured cathode cell based on hybrid material (HM) (**b**); 1–emitter; 2–gate. Block diagram of an optically controlled X–ray emitter array (**c**).

Modification of the emission structure [18] (see Figure 2a) by replacing the existing emitter with an HM structure and manufacturing electrodes from a light-transmitting indium tin oxide (ITO) alloy (see Figure 2b) made it possible to implement the array X-ray emitter design (see Figure 2c) for DCT systems. The performance of the device was ensured by the use of a high-speed laser scanner.

## 3. Statement of the Problem of Modeling Hybrid Material

Plasmon-resonant GNS are of particular interest as a new type of metal nanoparticles used in various technical and biomedical applications [19,20,21,22,29,30,31,32,33]. Compared to nanospheres, the advantage of their use is high absorption of radiation in the NIR (which is important, in particular, for laser medicine and biophotonics, including biosensing, optoporation, and the transfection of cells [19,29]. Compared to nanorods, GNS are characterized by a lower orientational and polarization sensitivity of induced optical [33,34,35] and temperature [36] fields. Below, we consider the possibility of intensifying the photoemission of electrons from composite photocathodes based on GNS embedded in a nanoscale film of diamond-like carbon.

**Figure 3:**
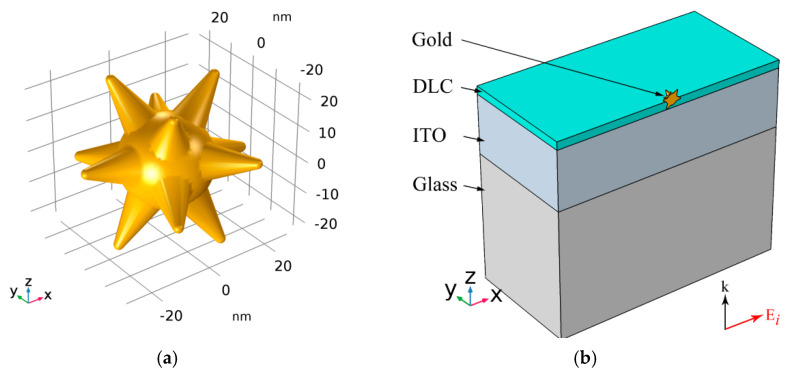
Geometric model of a nanostar (**a**) and a schematic representation of the computational domain (**b**) for finite element modeling of a nanostar embedded into a DLC film.

For the electrodynamic calculation of the optical characteristics of gold nanostars (see Figure 3a) in the medium, a 3D finite element model (shown in Figure 3b) was used. In the schematically shown computational domain, the wave equation for the electric field vector **E**:

(1) $$\nabla \times \mu_{r}{}^{-1}(\nabla \times E)-k_{\lambda }^{2}\varepsilon_{r}E=0$$

was solved. Here, μ_r_ and ε_r_ are the magnetic permeability and dielectric function, respectively, and *k*_λ_ = 2π/λ is the wavenumber of the irradiating light in vacuum. For all materials it was assumed: μ_r_ = 1, ε_r_ = (*n* − *ik*)^2^, where *n* and *k* are the real and imaginary parts of the complex refractive indices of materials, respectively.

At all interfaces of regions made of dissimilar materials, the boundary conditions of continuity of the tangential components of the strength and the normal components of the induction of the electric and magnetic fields were satisfied.

External surfaces modeling the transition to free space were constrained by PML «perfectly matched layers» [37,38]. The case of normal incidence of a plane wave in the positive direction of the *z*-axis with an electric vector directed along the *x*-axis was considered. For the magnetic field strength **H** in the symmetry plane *x*–*z* with the normal vector **y_0_**, the PMC («perfect magnetic conductor») boundary condition [37,38] was used:

**y_0_** × **H** = 0 (2)

A symmetric GNS model with spikes uniformly oriented along the angle is considered. The thermo-optical parameters of the hybrid material were simulated, taking into account the effect of limiting the electron path length in the spikes of the nanostar. As shown in ref. [31], this noticeably improves the agreement between theory and experiment.

The specific absorption power of radiation was calculated by the formula [21]:

(3) Q=νIm(εr)|E|2

where ν and **E** are the frequency and vector of the electric field strength, respectively.

To verify the model, the results of the calculation of the absorption and scattering cross-sections were compared with the experimentally determined spectral dependences of the extinction of a suspension of GNS in water and GNS on a glass plate [33]. A good quantitative agreement between the theoretical and experimental results was demonstrated. In particular, when using the two-fraction approximation of the GNS ensemble model in water, the coincidence of the first resonance peak of the normalized extinction spectrum near 900 nm was obtained in theory and the experiment. Additionally, the difference in the position of the second resonance peak near 1900 nm did not exceed 100 nm. The values of both peaks in theory and experiments coincided within a few percentage points. Therefore, the use of the described mathematical model seems reasonable.

Photocathodes of complex structure and shape are characterized by the localization of the concentration of photoexcited electrons in separate spatial regions, called “hot spots”. The estimation of the spatial distribution of the concentration of “hot” electrons in the CW irradiation mode was carried out within the framework of simplified formalism [20,21,22] based on the equation:

(4) $$\frac{d^{2}N(r)}{dsdE}=|E_{normal}(r){|}^{2}\frac{2e^{2}E_{F}^{2}}{\pi^{2}\gamma {\left(\hbar \omega \right)}^{4}}$$

where *d*^2^*N*(**r**)/*dsdE* is the number of hot electrons for unit area *s* and energy *E* in the vicinity of the considered point **r** on the surface, *E_normal_*(**r**) is the component of the complex amplitude of the electric field normal to the surface at point **r**, *e* is the absolute value of the electron charge, *E_F_* is the Fermi energy, *γ* is the rate for energy relaxation of single electrons, and *h*ν is the energy of the irradiating photons.

In accordance with the Ref. [21], the estimate of the influence of the spectral dependence of the function multiplied in Equation (4) by |*E_normal_*(**r**)|^2^ leads to the conclusion that this function can be approximated by a constant with an error of no more than 25%. Thus, for the analysis of the concentration distribution of hot electrons, the key characteristic is |*E_normal_*(**r**)|^2^, which is investigated in detail below.

The photoemission current density is also significantly determined by the electrostatic field, which lowers the height of the potential Schottky barrier at the metal-vacuum and DLC-vacuum interfaces. Calculations show that the spatial distribution of the electrostatic field in structures with field emission is significantly inhomogeneous [28,39]. Further, on the basis of numerical calculations, an analysis of the localization of the optical field (to identify areas of effective generation of hot electrons) and the spatial distribution of the electrostatic field (to intensify photoelectron emission) was carried out. The influence of the spatial separation of the regions of localization of the electrostatic field and hot electrons on the photoemission process is discussed.

## 4. Results of Modeling and Discussion

To describe the bulk dielectric functions of materials (gold [40], DLC [41], ITO [42]), the interpolated tabular data given in the above works were used. The dielectric constant of the glass substrate was taken to be ε_r_ = 2.25. The geometrical parameters of the GNS, which were used in the design of the computational model, were taken for certainty in accordance with the GNS, synthesized and experimentally investigated in ref [43]. Thermo-optical properties of such GNS have been studied theoretically earlier [34]. The results obtained, in particular, made it possible to explain the experimentally observed threshold nature of their photomodification when irradiated with nanosecond laser pulses. Therefore, the geometrical model of the GNS was a sphere 22 nm in diameter with twelve S-cone spikes with a height and base diameter of 10 nm, and a tip curvature radius of 1.5 nm.

**Figure 4:**
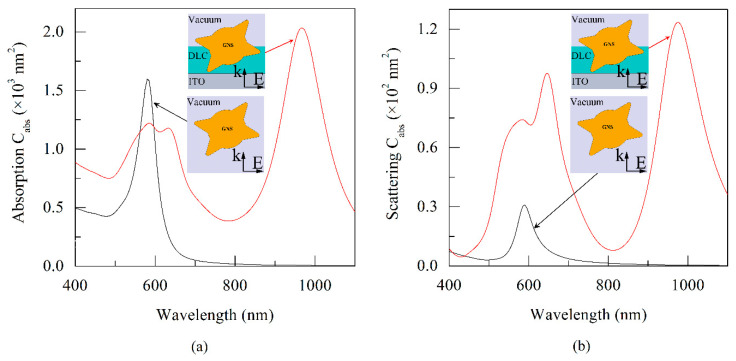
Spectral dependence of the absorption (**a**) and scattering (**b**) cross sections of a GNS in vacuum (shown schematically in the lower insert) and embedded in the GNS–DLC film (shown schematically in the upper insert). The inserts schematically show the position of the GNS in a vacuum (lower insert) and in a DLC film (upper insert) in a section of the structures by the *xz* symmetry plane.

From the calculations of the optical parameters of a GNS presented in Figure 4, it can be seen that in the case of GNS in a vacuum, the absorption and scattering cross-section spectra have one narrow resonance peak near λ = 600 nm. For GNS embedded in the HM structure, the shape of the spectra becomes much more complicated: the spectra noticeably broaden, and an additional pronounced resonance peak appears in the NIR near λ = 1000 nm.

It can be seen that halfway from the maximum, the width of the resonance curve of the absorption cross section increases by a factor of five, and taking into account the second peak, by a factor of nine. The appearance of this peak in the NIR was expected and is associated with an increase in the real part of the dielectric function of the medium surrounding the part of the GNS immersed in the DLC film [29,30]. Therefore, it can be expected that the main contribution to this shift is made by precisely those rays that are immersed in the DLC film. The non-trivial multi-peak shapes of the spectra are due to the fact that the GNS is only partially immersed in the DLC film. Various spikes, partially or completely immersed in a vacuum or in a film, mainly contribute to the short-wavelength or NIR region of the spectral curves, respectively.

**Figure 5:**
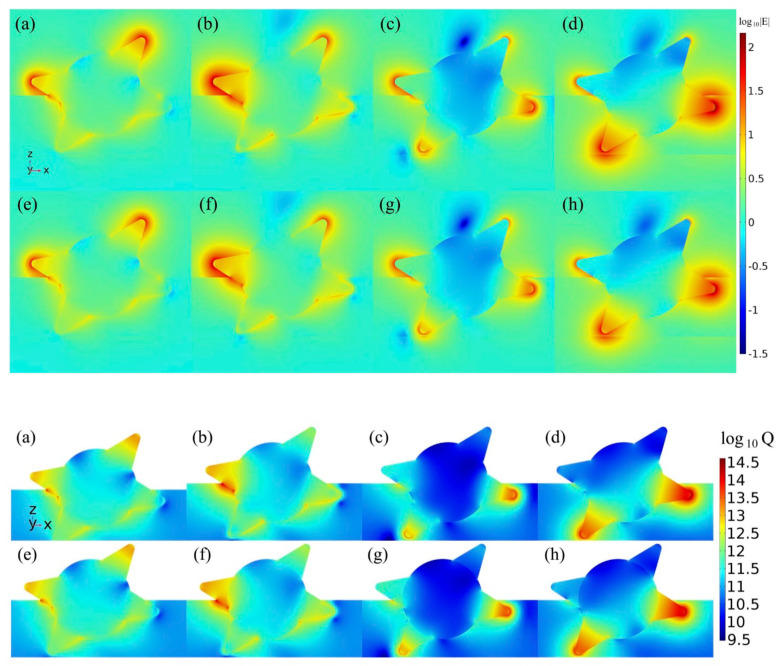
2D maps of normalized electric field (upper panel) and specific radiation absorption power *Q* under an irradiation field of intensity *I*_0_ = 1 W/cm^2^ (lower panel) in an HM slice at 584 nm (**a**,**e**); 634 nm (**d**,**f**); 808 (**c**,**g**); 970 nm (**d**,**h**) calculated for bulk (**a**–**d**) and size-corrected (**e**–**h**) dielectric function of spikes.

Slices in Figure 5 (upper panel) demonstrate how the localization zones of |*E*| are redistributed along different spikes of the GNS with a change of the laser wavelength. The relative change in the field amplitude, taking into account the size correction, is from 5% at 808 nm to 19% at 970 nm. In addition to the spike tips, localization zones are also observed on the exit/entry lines of the spikes into the DLC film. Therefore, in addition to the tips on the four spikes of the GNS, the field characteristics are analyzed at two points on the exit/entry lines of the spikes into the DLC film. The positions of these six points are shown in the inserts in the following figures. The regularities of localization of absorption zones are practically identical to those given for |*E*| above (see Figure 5 (lower panel)).

**Figure 6:**
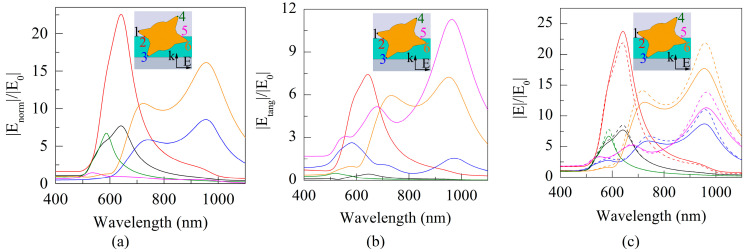
Spectral curves for modules of normal |*E_normal_*| (**a**), tangential |*E_tang_*| (**b**), and complete |*E*| (**c**) amplitudes of the electric field at six points on the GNS surface indicated in the insets. In panel (**c**), the solid and dotted curves were obtained with and without the size correction, respectively. The curves are normalized to the amplitude of the incident field |***E***_0_|. Field localization zones: points 1, 4 are the spike tips in vacuum; points 3, 6 are the spike tips in DLC; points 2, 5 are the exit/entry of the spikes into the DLC film. Curve colors match the point number colors.

The results in Figure 6 confirm the previously stated assumption about the cause of the multi-peak shape of the absorption and scattering cross sections. Namely, various spikes, partially or completely immersed in a vacuum or in a film, make the main contribution to the short-wave or NIR regions of the spectral curves, respectively. The difference between the solid and dashed curves in the fragment in Figure 5c, which reached 23% in the NIR, indicates the need to take into account the size correction to the dielectric function of the GNS spikes.

**Figure 7:**
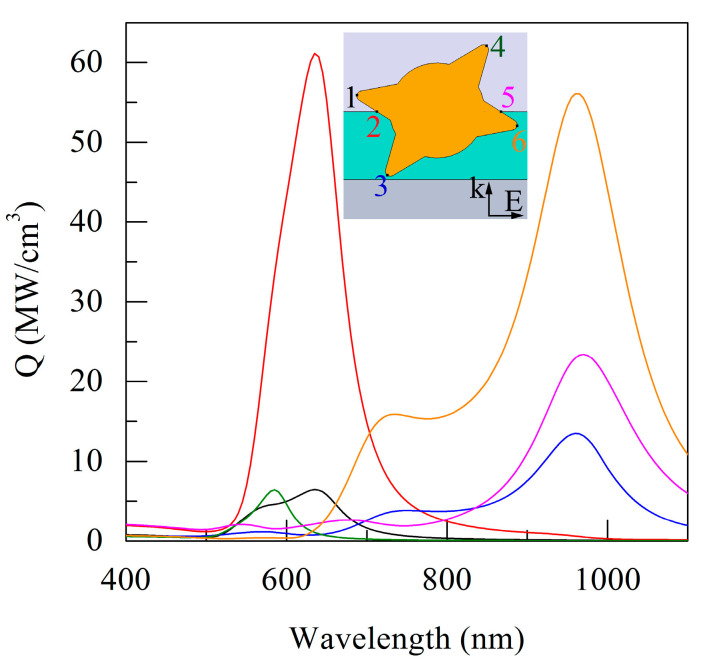
Spectral curves of the specific radiation absorption power *Q* at the indicated points on the GNS surface. The intensity of the irradiating field *I*_0_ = 1 W/cm^2^. The designations of points and curves are the same as in Figure 6.

The results of calculation of the specific power absorption *Q* at the selected points of the GNS are presented in Figure 7. It is possible to note the reduced values of *Q* at the tips of the GNS spikes, which are predominantly in a vacuum compared to other GNS spikes immersed in the DLC. This difference in *Q* value can reach 3–9-fold. It is characteristic that the spectral curves *Q* for each of the points are predominantly single-peaked.

**Figure 8:**
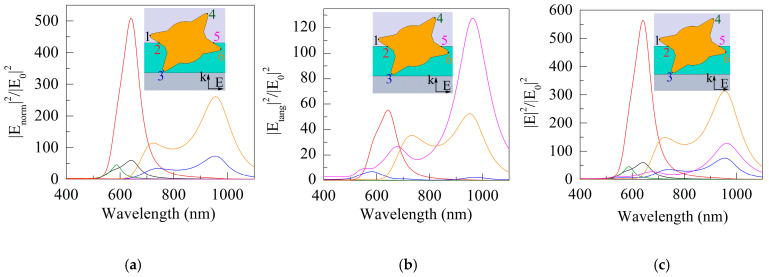
Spectral curves for the squares of the modules of normal |*E_normal_*| (**a**), tangential |*E_tang_*| (**b**) field components and total field (**c**) normalized to the square of the modulus of the irradiating field |*E*_0_| at six specified points on the GNS surface. The designations of points and curves are the same as in Figure 6.

Figure 8a shows the spectral dependences of the square of the modulus of the normal component |*E_normal_*|, normalized to the square of the modulus of the irradiating field strength |*E*_0_| and proportional to the concentration of hot electrons [18,19,20]. Note that despite the symmetric position of points 1 and 6 with respect to the electric vector of the irradiating field, the ratio ψ = |*E_normal_*|^2^/|*E*_0_|^2^ for them differs by a factor of five. Obviously, the reason for this is the influence of environmental properties. If point 1 belongs to the tip of the spike directed into vacuum, then point 6 is on the tip of the spike partially immersed in the DLC film. In this case, ψ at point 6 (in the DLC film) is higher than ψ at point 1 (in vacuum). Thus, one should expect an increased concentration of hot electrons in the spike with point 6, which favors the injection of electrons into the near-surface DLC layer and their further tunneling into the vacuum.

Comparison of the results in Figure 8a,b indicates the prevalence of the normal component of the field over the tangential for all studied points, except for point 5, in which the situation is directly opposite. Therefore, the spectral curves of the total field in Figure 8c are also qualitatively close for all points, except for the spectrum of point 5.

**Figure 9:**
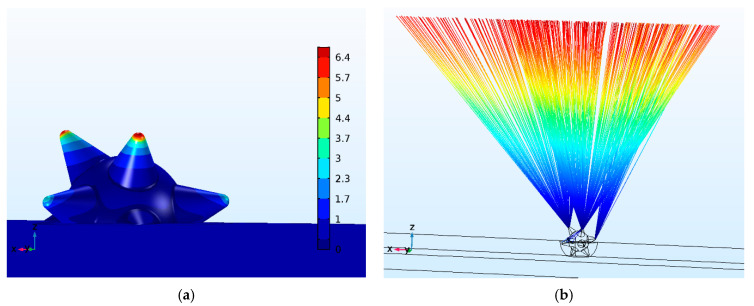
A map of the gain distribution ξ_F_ of the electrostatic field on the surface of GNS embedded into the DLC film and its surroundings (**a**), the vector of an external uniform field with a strength *F*_0_ is directed along the *z*-axis; results of modeling the trajectories of electrons emitted from the particular points of GNS spikes (**b**).

To assess the role of the electrostatic field in stimulating the photoemission of a HM based on GNS-DLC, it is important to know the features of the localization of the field under the influence of external high voltage. The greatest interest in this respect is the information about the concentration (amplification) of the external field strength *F*_0_ on the inhomogeneities of the GNS form embedded in the DLC. The results of modeling the distribution of this gain ξ_F_ = |*F*(**r**)|/|*F*_0_| (*F*(**r**) is the vector of the electrostatic field strength at the current point with the radius vector **r**) are shown in Figure 9a. It can be seen that the orientation of the rays relative to the external field vector *F*_0_ significantly affects the distribution of ξ_F_. Local maxima ξ_F_ are observed at the points of the GNS rays, but the value of each of the maxima increases with a decrease in the angle between the spike axis and the direction of the vector *F*_0_. Thus, for a spike with a minimum deviation from the direction of the vector *F*_0_, the value of ξ_F_ is maximum and equal to 6.7. For a spike with a maximum deviation from the direction of the vector *F*_0_, the value of ξ_F_ falls twice, reaching a value of 3.3. It should be noted that, according to the calculation, the external field *F*_0_ also has a positive effect on the conditions of injection of hot electrons into the DLC film. Therefore, on the tip of the GNS spike, slightly deepened in the film (in Figure 9 it is designated as point 6), the field gain is 2.

Hot electrons formed in the above-mentioned localization zones of the normal component of the *E_normal_* optical field have a high probability of tunneling through the Schottky barrier in strictly limited areas of the concentration of the electrostatic field. From the comparative analysis of the results in Figure 5a (upper panel), Figure 8a and Figure 9a, it follows that in the case under consideration, such localized zones of the coordinated effect of optical and electrostatic fields should be recognized primarily in the vicinity of points 1 and 4. This is due, firstly, to the high efficiency of conversion the laser beam energy into hot electrons (see Figure 8a) and, secondly, to the relatively high electrostatic field gain (see Figure 9a). Additional consideration of the influence of the plasmon effect as well (see Figure 8c) allows us to conclude that in the vicinity of the tips of both spikes (points 1 and 4), the conditions for the generation of hot electrons are approximately equivalent. However, there is a difference between them in the magnitude of the amplification of the electrostatic field. At point 4, ξ_F_ is twice as high as at point 1. This will affect the magnitude of the lowering of the Schottky barrier and, as a result, will lead to more intense tunneling of hot electrons into vacuum. The results of modeling the trajectories of photoelectrons from the tips of the GNS spikes are shown in Figure 9b. Under conditions of uniformly accelerated motion in a uniform electrostatic field, this beam will form a spot with a diameter of no more than 60 µm on the anode located at a distance of 20 mm from the emitter.

The high efficiency of generating hot electrons in the vicinity of points 2 and 6 (see Figure 5a (upper panel), Figure 8a and Figure 9a,c) triggers an alternative mechanism—injecting electrons into the DLC film, transporting them to the film surface and further emission into the vacuum, as discussed in refs [15,17]. The lowest degree of matching of the optical and electrostatic fields in the vicinity of points 3 and 5 makes the probability of both generation and successful transport of hot electrons into a vacuum low.

## 5. Conclusions

A new hybrid material based on GNS embedded in a nanoscale DLC film has been proposed and its properties have been theoretically investigated. The results obtained make it possible to assess the prospects of using the effect of an increased concentration of hot electrons in nanostructures with plasmonic materials for photostimulated field emission of electrons and possible applications in the field of biomedical instrumentation and sensing. In particular, the material is promising in the development of a matrix source with photoemission for X-ray computed tomography sensors, providing an increase in resolution and a decrease in the criticality of local heating during tunneling electron emission of an X-ray gun with a matrix cathode.

The broadband effect of optical excitation of the hybrid material in the visible and NIR wavelength ranges is noted. The areas of localization of electrostatic and optical fields in GNS, in which their effective matching is ensured, are determined.
